# Supplementary material for: Psychometric properties of the Chinese version of the Resilience Scale (RS-14): Preliminary results
Source: PLoS One. 2020 Oct 30;15(10):e0241606. doi: 10.1371/journal.pone.0241606 (PMC7598507; doi:10.1371/journal.pone.0241606)
Supplement: Attachment — Submitted filename: Response to Reviewers.docx [file pone.0241606.s002.docx]

Thank you, editor.

“Before sending out your manuscript for external review, I ask you to specify the estimation method that was used in CFA.”

Response: We added the estimation method in paper. According to the skewness and kurtosis, the maximum likelihood (ML)estimation was selected in CFA.

Furthermore, I wonder why you explained skewness and Kurtosis a lot but you reported nothing about skewness and Kurtosis in the results. Please write a sentence at least.

Response: We added the related instructions in the results.

“All the skewness and kurtosis statistics were within the acceptable range (skewness within ±3.00 and kurtosis within ±8.00 which indicating normally distributed” .
